# Neuroptera of Canada

**DOI:** 10.3897/zookeys.819.26683

**Published:** 2019-01-24

**Authors:** David C.A. Blades

**Affiliations:** 1 Research Associate, Royal British Columbia Museum, 675 Belleville St, Victoria, BC, V8W 9W2, Canada Royal British Columbia Museum Victoria Canada

**Keywords:** antlion, aphidlion, biodiversity assessment, Biota of Canada, lacewing, mantidfly, Neuroptera, owlfly

## Abstract

The Neuroptera of Canada consists of 101 extant species, an increase of 26 (35%) since the previous assessment of the fauna in 1979. More than 48 additional species are believed to occur in Canada based largely on recent DNA evidence and new distribution records. The Barcode Of Life Data System (BOLD) currently includes 141 Barcode Index Numbers (BINs) for Canadian Neuroptera. Canadian fossils have thus far yielded 15 species in three families of Neuroptera.

The order Neuroptera, including the lacewings, antlions, owlflies and relatives, contains approximately 6400 extant species worldwide (Oswald and Machado 2018), and approximately 350 in America north of Mexico (Penny et al. 1997). As of 2017, the Canadian fauna consists of 101 extant species, an increase of nearly 35% since the previous assessment by Kevan (1979) (Canadian Endangered Species Conservation Council 2016) (Table 1).

The significant increase in species known from Canada since 1979 is a result of research concentrated on the taxonomy and faunistics of Canadian species. Most of the research has focused on the most speciose familes (Table 1), the Hemerobiidae (Klimaszewski and Kevan 1985, 1987a, b, 1988, 1989, 1992, Kevan and Klimaszewski 1986, Oswald 1988, 1993, Klimaszewski et al. 2009) and the Chrysopidae (Adams and Garland 1981, Garland 1984, 1985, 2000a, b, Tauber 2003, Penny 2006, Garland and Kevan 2007). Other significant works include the review of the Mantispidae of Canada by Cannings and Cannings (2006), the Neuroptera of North America by Penny et al. (1997), and the neuropteroid insects of British Columbia by Scudder and Cannings (2009). As of 2017, more than half of Canadian species have been DNA barcoded and have Barcode Identification Numbers (BINs) (Ratnasingham and Hebert 2013) associated with them (Table 1).

Two of the more notable additions to the Canadian fauna are *Nallachiusamericanus* (McLachlan) (Dilaridae) and *Ululodesquadripuntatus* (Burmeister) (Ascalaphidae) (Garland and Marshall 1980, Preney and Jones 2017). Each of these species belong to families previously unrecorded in Canada, but were predicted by Kevan (1979) to occur here. The mantidfly, *Dicromantispasayi* (Banks) (Mantispidae) is another species that was expected by Kevan (1979) and subsequently recorded in southern Ontario (Cannings and Cannings 2006). The chrysopid, *Ninetagravida* (Banks) was rediscovered on Canada’s Pacific coast after 90 years (Garland 2000b), while the ithonid, *Polystoechotespunctatus* (Fabricius) appears to be extirpated from the eastern half of North America having not been recorded there since 1952 (Marshall 1996).

Most of the recent increase in species known from Canada has been in the families Coniopterygidae and Hemerobiidae (Table 1). Very little work has been done on the North American Coniopterygidae since Meinander (1974) revised the family (Penny et al. 1997, Meinander et al. 2009). Given their small size and cryptic nature it is likely that research on the Coniopterygidae will yield at least a few new species and distribution records for Canada. BINs for specimens of Coniopterygidae suggest that there may be 20 or more species in this family left to describe. BINs also indicate that there may be several undescribed species of Hemerobiidae, Chrysopidae, and Mymeleontidae.

Hemerobiidae represents a distinctly northern and western group in North America and is the most species-rich family of Neuroptera in Canada with 43 species (70% of the North American hemerobiid fauna) (Table 1). Kevan and Klimaszewski (1986) characterized the Canadian fauna as “boreo-alpine”. Several species are transcontinental in Canada, their ranges extending south into the northernmost parts of the eastern US and along the mountain ranges of the western US. Kevan and Klimaszewski (1986) describe five general distribution patterns for the Hemerobiidae: Holarctic, transcontinental Nearctic, western Nearctic, disjunct Nearctic (western with scattered eastern records) and eastern Nearctic. One species, *Wesmaeliuslongipennis* (Banks), was listed by Kevan and Klimaszewski (1986) as endemic to California. However, specimens of *W.longipennis* in the Canadian National Collection, collected from coastal and interior locations in British Columbia, and one from Quebec, in the 1920s and 1930s, were recently identified (J Klimaszewski pers. comm.). This is a significant extension of the known range and implies that the California records may represent the southern extent of a more widely distributed northern species.

Few Neuroptera in Canada are considered to be exotic species. These introductions include three Coniopterygidae (*Conwentziapsociformis* (Curtis), *Semidalisvicina* (Hagen), *S.pseudouncinata* Meinander), three Hemerobiidae (*Psectradiptera* (Burmeister), *Wesmaeliussubnebulosus* (Stephens), *Micromusvariegatus* (Fabricius), and the chrysopid, *Chrysoperlacarnea* (Stephens) (Meinander 1972, Kevan and Klimaszewski 1986, Meinander et al. 2009). *Chrysoperlacarnea*, once considered to be of Eurasian origin and introduced to North America (Henry 1983, Brooks 1994) is mass-produced and introduced into agricultural systems as a biocontrol agent (Tauber et al. 2000). *Chrysoperlacarnea* was recently divided into a complex of 15 or more species that are reproductively isolated by their mate-attraction songs (Henry et al. 2011) and analysis of commercially produced specimens labeled as *C.carnea* were in fact dominated by *C.plorabunda* (Fitch) (a North American species) and no *C.carnea* were present (Henry and Wells 2007).

**Table 1.** Census of Neuroptera in Canada.

**Taxon^1^**	**No. species reported in Kevan (1979)**	**No. species currently known from Canada^2^**	**No. BINs^3^ available for Canadian species**	**Est. no. undescribed or unrecorded species in Canada**	**General distribution by ecozone^4^**	**Information sources**
Coniopterygidae	7	12 (3)	56	>20	all except Arctic	Meinander 1972, Penny et al. 1997, Canadian Endangered Species Conservation Council 2016
Sisyridae	3	3	3	0	all ecozones	Penny et al. 1997, Bowles, 2006
Dilaridae	0	1	0	0	Mixedwood Plains	Prenny and Jones 2017
Berothidae	1	1	1	2	Western Interior Basin	Scudder and Cannings 2009
Mantispidae	3	4	4	0	Montane Cordillera, Western Interior Basin, Prairies, Mixedwood Plains	Cannings and Cannings 2009
Hemerobiidae	28	43 (3)	43	15	all ecozones	Kevan and Klimaszewski 1986, Penny et al. 1997, Canadian Endangered Species Conservation Council 2016
Chrysopidae	25	26 (1)	21	6	all except Arctic	Garland and Kevan 2007, Canadian Endangered Species Conservation Council 2016
Ithonidae ^5^	1	1	1	0	Montane Cordillera, Western Interior Basin, Prairies, Mixedwood Plains^6^	Penny et al. 1997
Ascalaphidae	0	1	0	0	Mixedwood Plains	Garland and Marshall 1980
Myrmeleontidae	7	9	12	>5	most ecozones south of taiga	Penny et al. 1997, Canadian Endangered Species Conservation Council 2016
**Total**	**75**	**101 (7)**	**141**	**>48**		

**^1^** Classification follows the phylogeny indicated in Engel et al. (2018). **^2^**Numbers in parentheses indicate number of non-native species included in the total. **^3^**Barcode Index Number, as defined in Ratnasingham and Hebert (2013) ^4^See figure 1 in Langor (2019) for a map of ecozones. **^5^**Ithonidae now includes the Polystoechotidae (Engel et al. 2018). **^6^**Believed to be extirpated from eastern North America (Marshall 1996).

Future research on Canadian Neuroptera is likely to yield some new species and range extensions in the more diverse families (Table 1). The Paleobiology Database (http://fossilworks.org) indicates that the fossil record has thus far yielded 15 species from three families for Canada, and 68 species in five families for North America, and research on Canadian fossil deposits may reveal additional species. Other interesting avenues of research include the application of native Neuroptera as control agents in agricultural settings, the mating songs of *Chrysoperla* and the existence of this phenomenon and other mate selection methods in related taxa and systematic revisions of the Myrmeleontidae and Coniopterygidae.
